# Ni–Cu High-Loaded Sol–Gel Catalysts for Dehydrogenation of Liquid Organic Hydrides: Insights into Structural Features and Relationship with Catalytic Activity

**DOI:** 10.3390/nano11082017

**Published:** 2021-08-06

**Authors:** Yuliya Gulyaeva, Maria Alekseeva (Bykova), Olga Bulavchenko, Anna Kremneva, Andrey Saraev, Evgeny Gerasimov, Svetlana Selishcheva, Vasily Kaichev, Vadim Yakovlev

**Affiliations:** Federal Research Center Boreskov Institute of Catalysis, Akad. Lavrentiev Ave. 5, 630090 Novosibirsk, Russia; obulavchenko@catalysis.ru (O.B.); kremneva@catalysis.ru (A.K.); asaraev@catalysis.ru (A.S.); gerasimov@catalysis.ru (E.G.); svetlana@catalysis.ru (S.S.); vvk@catalysis.ru (V.K.); yakovlev@catalysis.ru (V.Y.)

**Keywords:** dehydrogenation, solid solution, nickel–copper catalyst, LOHC

## Abstract

The heightened interest in liquid organic hydrogen carriers encourages the development of catalysts suitable for multicycle use. To ensure high catalytic activity and selectivity, the structure–reactivity relationship must be extensively investigated. In this study, high-loaded Ni–Cu catalysts were considered for the dehydrogenation of methylcyclohexane. The highest conversion of 85% and toluene selectivity of 70% were achieved at 325 °C in a fixed-bed reactor using a catalyst with a Cu/Ni atomic ratio of 0.23. To shed light on the relationship between the structural features and catalytic performance, the catalysts were thoroughly studied using a wide range of advanced physicochemical tools. The activity and selectivity of the proposed catalysts are related to the uniformity of Cu distribution and its interaction with Ni via the formation of metallic solid solutions. The method of introduction of copper in the catalyst plays a crucial role in the effectiveness of the interaction between the two metals.

## 1. Introduction

In a view of the obvious focus on clean and resource-saving (sustainable) energy, the use of hydrogen as an energy carrier seems inevitable in the long term. The main problem restricting the development of hydrogen energy is the lack of technologies compatible with existing infrastructure for the safe storage and transportation of hydrogen. One of the promising approaches to hydrogen storage and transport is the use of liquid organic hydrogen carriers (LOHCs) [1,2,3]. In these structures, hydrogen can be stored by forming a chemical bond, and its reverse release should be thermodynamically favorable. Herein, the LOHC concept is based on the use of chemical compounds susceptible to reversible hydrogenation and dehydrogenation, such as cyclic hydrocarbons (for example, benzene/cyclohexane, toluene/methylcyclohexane, or biphenyl/bicyclohexyl) or heterocyclic compounds (carbazoles, pyridines, quinolones, pyrroles, etc.). Importantly, the viability of the proposed technology is greatly determined by the development of catalysts with high activity, selectivity, and stability in the endothermic dehydrogenation half-cycle. Therefore, the catalyst design should ensure high selectivity, avoiding adverse reaction routes due to the rupture of carbon–carbon and carbon–heteroatom bonds [4,5]. In addition, it is important for the dehydrogenation catalyst to be resistant to coke deposition resulting in the deactivation of the catalyst [6]. Traditionally, catalysts based on noble metals (mainly Pt and Ru) have been proposed for both hydrogenation and dehydrogenation routes [7,8,9,10]. Supported Pt-based catalysts are known to be active in the hydrogenation of both linear unsaturated hydrocarbons and aromatic compounds—for example, benzene to cyclohexane—in the production of adipic acid [11]. Commercial Ru/Al_2_O_3_ catalysts have been shown to effectively catalyze the hydrogenation of methyl-diphenylbenzene [1]. As for the dehydrogenation of cycloalkanes, the most widely used catalysts are Pt-containing catalysts promoted with Re, Ni, Sb, Au, and/or other metals [12,13,14]. Chiyoda Corporation proposed a hydrogen-storage technology using the LOHC pair “toluene (TOL)–methylcyclohexane (MCH)” and a Pt/Al_2_O_3_ catalyst modified with sulfur [15,16]. However, the method of introduction of sulfur was not disclosed due to commercial secrecy, and it remains unclear what happened to the catalyst in the absence of a sulfur-containing source during the process. Other noble-metal-based catalysts have not achieved similar levels of practical use in hydrogen storage.

In general, the high cost of noble metals, and sometimes their insufficient selectivity in the target dehydrogenation reaction, prompts reduction of the use of precious metal catalysts by the introduction of a second metal (Ni, Sn, Mn, etc. [9,17,18,19,20,21]) and the search for alternative catalytic systems. In particular, non-precious monometallic catalysts based on Ni, Mo, Cu, Ag, Zn, and Sn have been proposed [22,23,24,25]. The widely available nickel-based catalysts proved to be highly promising. Nevertheless, monometallic Ni-based catalysts were shown to possess higher activity but extremely low selectivity and high coking tendency during the dehydrogenation of MCH [26,27]. A significant increase in selectivity toward benzene was achieved for the dehydrogenation of cyclohexane using bimetallic Ni–Cu/SiO_2_ catalysts [27]. Although the structure of bimetallic compounds was not studied in detail, the formation of a Ni–Cu solid solution could be assumed to be the reason for the effective suppression of untargeted C–C bond cleavage. In general, the information related to the effect of doping elements and the structure of bimetallic Ni–Cu catalysts on their performance in the dehydrogenation of MCH is scarcely represented in the literature. Thus, the lack of discussion provided in the references concerning a deep understanding of such effects became a driving force for the present study.

Here we propose a new type of Ni-based catalyst synthesized via the heterophase sol–gel technique. To the best of our knowledge, this approach has never been implemented for heterogeneous systems catalyzing dehydrogenation reactions. The catalysts were additionally modified by a second metal—copper—and studied in the dehydrogenation of MCH. A special focus has been placed on the effects of the copper-to-nickel ratio and the method of introduction of copper on the activity and selectivity of the catalysts in the target reaction. The catalysts were studied via X-ray diffraction (XRD), high-resolution transmission electron microscopy (HRTEM), energy-dispersive X-ray (EDX) analysis, X-ray photoelectron spectroscopy (XPS), X-ray absorption spectroscopy (XAS), temperature-programmed reduction with H_2_ (TPR-H_2_), and N_2_ and CO adsorption techniques. In addition, several chosen catalytic systems were studied in situ via XRD and XAS in a hydrogen-containing atmosphere in order to observe the effect of temperature on the formation of the active component of the catalyst and its structural features.

## 2. Materials and Methods

### 2.1. Chemicals

All chemicals were commercially available and used without additional purification. For catalyst synthesis: nickel(II) carbonate basic hydrate (≥98%), copper(II) carbonate basic (≥98%), copper(II) nitrate hydrate (≥98%), aqueous ammonia (99%), and ethyl silicate (≥99%) were supplied by the joint-stock company Reakhim JSC (Staraya Kupavna, Russia). For catalyst testing, methylcyclohexane (99%) was supplied by Acros Organics (Fair Lawn, NJ, USA). Hydrogen (5.0) and argon (6.0) gases were purchased from Pure Gases Ltd. (Novosibirsk, Russia).

### 2.2. Catalyst Preparation

Two approaches based on the heterophase sol–gel method [28,29] were used for the catalyst preparation; SiO_2_ was used as a stabilizing agent in both cases (15–20 wt% of total content of the catalysts in an oxide form). According to the first approach, appropriate amounts of solid nickel(II) carbonate basic hydrate and copper(II) carbonate basic were mixed with required amounts of an aqueous solution of ammonia (25% of NH_3_) and distilled water. The copper precursor was not introduced in the case of a monometallic nickel catalyst. Then, ethyl silicate (ES, with SiO_2_ content of 32 wt%) was added to the mixture. The obtained suspension was dried in air overnight at 100 °C and then calcined at 400 °C for 4 h. According to the second approach, which was first implemented in the present study, the copper precursor was introduced via wetness impregnation of the Ni–SiO_2_ system using an aqueous solution of copper (II) nitrate hydrate. All other calcination procedures were the same as in the first approach. Before characterization, the catalysts were reduced in a quartz tubular reactor with a hydrogen flow of 500 mL min^−1^ (0.1 MPa) at 400 °C for 1 h, followed by cooling to room temperature (ex situ reduction). Thereafter, the catalysts were purged with an argon flow and passivated with ethanol. The catalysts were referred to as “passivated”.

The catalysts prepared according to the first and second approaches were respectively designated as CuXNiY-SiO_2_ and CuX/NiY–SiO_2_, where X and Y correspond to the nominal percentages of nickel and copper relative to the total amount of metals (wt%). The whole series of the catalysts prepared according to the first approach was denoted as “SG series”, while the second series was denoted as “pCu series”. For comparison, the monometallic Ni–SiO_2_ system was also prepared. The chemical compositions for the respective catalyst series are listed in Table 1, as well as the atomic copper-to-nickel and silicon-to-nickel ratios.

Importantly, in the case of the pCu series, the maximum Cu/Ni atomic ratio was 0.4. For the SG series, the same could not be attained, because it was noticed that a high amount of the water-insoluble Cu-containing precursor negatively affects the formation of a final sol–gel product. This negative effect was most likely associated with a low surface area of the Cu-containing reagent. Therefore, providing a Cu loading of about 30 wt% in the SG series did not make much sense. Based on the synthesis approach, the Si/Ni atomic ratio was constant for the pCu catalysts, while it gradually increased from 0.33 to 0.41 with copper loading for the SG samples.

### 2.3. Catalyst Testing in the Dehydrogenation of MCH

The dehydrogenation of MCH was performed in a fixed-bed flow reactor in a temperature range of 250–300 °C at ambient pressure (0.1 MPa). Typically, 0.5 g of catalyst (0.25–0.5 mm fraction) was loaded in the reactor and diluted with quartz sand of the same fraction to a volume of 3 cm^3^. Before the experiment, the catalyst was reduced inside the reactor, following the same procedure described in the preparation section. Thus, the catalyst samples were activated in a hydrogen flow of 500 cm^3^ min^−1^ at 400 °C for 1 h, after which the reactor was cooled down to 250 °C. For testing, MCH was fed to the reactor by a liquid pump with a flow rate of 12 cm^3^ h^−1^. An equimolar mixture of hydrogen and argon (total flow of 400 cm^3^ min^−1^) was supplied to the reactor. The outlet gas components were analyzed using a Chromos-GC chromatograph (Chromos Engineering, Dzerzhinsk, Russia) equipped with two columns packed with a molecular sieve or activated carbon (2-mm inner diameter and 3-m length), with thermal conductivity and flame ionization detectors, respectively. Liquid products were collected in a condensation system and analyzed using an Agilent Technologies 7820A chromatograph (Agilent Technologies, Inc., Santa Clara, CA, USA) equipped with a capillary column (polyethylene glycol, 30-m length, 0.25-mm inner diameter, 0.25-μm film thickness) and a flame ionization detector. The performance of each catalyst was evaluated based on the MCH conversion (X_MCH_), selectivity to TOL (S_TOL_), and yield of TOL (Y_TOL_), according to the Equations (1)–(3), respectively. These calculations were performed taking into account the amount of methane formed during the process:

(1) $$X_{MCH}\left(\%\right)=\frac{n_{MCH}^{0}-n_{MCH}^{fin}}{n_{MCH}^{0}}\cdot 100$$

(2) $$S_{TOL}\left(\%\right)=\frac{n_{TOL}}{n_{MCH}^{0}-n_{MCH}^{fin}}\cdot 100$$

(3) $$Y_{TOL}\left(\%\right)=\frac{n_{TOL}}{n_{MCH}^{0}}\cdot 100=\frac{X_{MCH}\cdot S_{TOL}}{100}$$

where $n_{MCH}^{0}\;$is the initial amount of MCH (mol), $n_{MCH}^{fin}$ is the final amount of MCH (mol) in the reaction mixture, and $n_{TOL}$ is the amount of TOL (mol) in the liquid phase after the reaction.

### 2.4. CO Chemisorption

The CO chemisorption was measured in pulse mode using a Chemosorb analyzer (Modern Laboratory Equipment, Novosibirsk, Russia) equipped with a thermal conductivity detector. Fifty milligrams of catalyst was loaded into a U-shaped quartz reactor and treated in a H_2_ flow (100 cm^3^ min^−1^) upon heating to 400 °C, with a heating rate of 10 °C min^−1^. The reactor was kept at the final temperature for 20 min, then purged with argon, and then cooled to ambient conditions. After cooling, pulses of CO (0.1 cm^3^) were fed to the reactor until the outlet amount of CO stopped changing. Then, the amount of chemisorbed CO was estimated.

### 2.5. Nitrogen Physisorption

Textural properties of the catalysts were determined by N_2_ adsorption using an ASAP-2400 volumetric adsorption apparatus (Micromeritics Instrument Corp., Norcross, GA, USA). Prior to analysis, the catalysts were outgassed at 150 °C in vacuum at 0.13 Pa for 4 h. The specific surface area (SSA) was calculated via the Brunauer–Emmett–Teller method, using nitrogen adsorption isotherms measured at −196 °C.

### 2.6. Temperature-Programmed Reduction

For all TPR-H_2_ experiments, a catalyst in the oxide state (0.1 g) was placed in a U-shaped quartz reactor and heated to 800 °C, with a constant heating rate of 6 °C min^−1^ in a 10 vol% H_2_/Ar flow (20 cm^3^ min^−1^). The hydrogen consumption was measured with a thermal conductivity detector.

### 2.7. X-ray Diffraction

The phase composition of samples was studied using a D8 Advance X-ray diffractometer (Bruker, Germany), using monochromatic Cu-Kα radiation (λ = 1.5418 Å). The diffractometer was equipped with a Bruker LynxEye 1D detector (Bruker, Germany), which allowed us to obtain a diffraction pattern in the 2θ range from 20° to 80°, with a step of 0.05°. A mean size of the coherent scattering domain (CSD) was determined according to the broadening of reflections using the Scherrer formula. Cell parameters of the metallic Ni phase were calculated using the Polycrystal software package based on 111 and 220 reflections of Ni [30].

In addition, the catalysts were analyzed during in situ reduction in an XRK-900 high-temperature chamber (Anton Paar, Graz, Austria) with a flow of 10 vol% H_2_ in Ar (100 cm^3^ min^−1^). A catalyst was heated stepwise at a rate of 12 °C min^−1^ up to 150, 200, 300, 400, and 500 °C, and at each temperature, the XRD patterns were recorded.

### 2.8. High-Resolution Transmission Electron Microscopy

The morphology and microstructure of the catalysts were studied via transmission electron microscopy. The images were obtained using a JEM-2010 electron microscope (JEOL Ltd., Tokyo, Japan) operated at an accelerating voltage of 200 kV and a lattice resolution of 0.14 nm. Additionally, the structure of the Cu20/Ni80–SiO_2_ catalyst was studied using a Themis Z electron microscope (Thermo Fisher Scientific, Eindhoven, The Netherlands) equipped with a Ceta 16 CCD sensor and a corrector of spherical aberrations, which provided a maximum lattice resolution of 0.07 nm at an accelerating voltage of 200 kV. The microscope was also equipped with an EDX Super-X spectrometer (Thermo Fisher Scientific, Eindhoven, The Netherlands) with a semiconductor Si detector providing an energy resolution of 128 eV. The samples for the HRTEM study were deposited on a holey carbon film mounted on an aluminum grid by the ultrasonic dispersal of the catalyst suspension in ethanol.

### 2.9. X-ray Photoelectron Spectroscopy

The XPS spectra were measured using a photoelectron spectrometer (SPECS Surface Nano Analysis GmbH, Berlin, Germany) equipped with an XR-50M X-ray source and a PHOIBOS 150-MCD-9 hemispherical electron energy analyzer. Before XPS analysis, the catalysts were additionally reduced in hydrogen at 350 °C; for 30 min at atmospheric pressure in a special high-pressure cell connected directly to the spectrometer. After that, the catalyst was vacuumed, cooled to room temperature, and transferred to the analytical chamber of the spectrometer without contact with air. Such an approach allows us to “freeze” the reduced state and analyze it via XPS [31].

The core-level spectra were obtained using the monochromatic Al-Kα radiation (hν = 1486.74 eV) in a fixed-pass energy mode under ultrahigh-vacuum conditions. The charge shift was corrected by setting the Si*2p* peak at 103.5 eV. Curve fitting was performed using CasaXPS software [32]. The line shape used in the fit was a symmetric function obtained by a summation of the Gauss and Lorentz functions. A Shirley-type background was subtracted from each spectrum. Relative element concentrations were determined from the integral intensities of the core-level spectra using the photoionization cross-sections, as described by Scofield [33].

### 2.10. X-ray Absorption Spectroscopy

X-ray absorption spectra at the K-edges of Ni and Cu were obtained at the Structural Materials Science Beamline at the Kurchatov Synchrotron Radiation Source (National Research Center “Kurchatov Institute”, Moscow, Russia). The beamline is described in detail elsewhere [34]. The electron storage ring was operating at 2.5 GeV in decay mode, with a top current of 150 mA. The synchrotron radiation was monochromatized using a single Si(111) crystal in the form of a monoblock with a notch mounted on a goniometric head. All X-ray absorption spectra were obtained in pass-through geometry. To perform the XAS studies, powder samples were mixed with amorphous BN and pressed into thin, self-supported pellets (7 mg of catalyst and 100 mg BN; dilution of approximately 1:14). For in situ experiments, the pellets were placed in a high-temperature cell, heated to 400 °C in a flow of 10 vol% H_2_ in nitrogen, and treated under these conditions for 20 min. The X-ray absorption spectra of the reference samples (metallic Ni and Cu foils) were recorded simultaneously with a spectrum of an analyzed sample for the calibration of the monochromator. The ionization currents were measured with Keithley 6487 digital picoamperemeters (Keithley Instruments LLC, Cleveland, OH, USA). The obtained spectra were processed according to standard procedures with the IFEFFIT software package [35]. The threshold ionization energy E_0_ was determined from the maximum of the first derivative of the experimental curves near the K-edge.

### 2.11. CHNS Analysis of Catalysts after Dehydrogenation of MCH

The CHNS elemental analysis of spent catalysts was performed using an Elementar Vario EL Cube analyzer (Elementar Analysensysteme GmbH, Langenselbold, Germany). All measurements were carried out at least in triplicate, and average values are reported.

## 3. Results and Discussion

### 3.1. MCH Dehydrogenation

All of the catalysts showed high activity in the dehydrogenation of MCH within a temperature range of 250–350 °C. The main differences in the catalytic behavior were observed above 300 °C, where the formation of the target product—toluene (TOL)—was complicated by the increasing contribution of side reactions. Hence, the hydrogenolysis of the C_arom_–CH_3_ bond of toluene with the formation of methane and benzene became especially noticeable at elevated temperatures. Table 2 summarizes the catalytic data obtained for MCH conversion, selectivity toward TOL, and yields of TOL measured at 325 °C. A detailed analysis of the observed experimental tendencies is described elsewhere [36]. As seen in Table 2, the monometallic nickel-based catalyst exhibited the lowest selectivity toward TOL formation among the catalysts tested, which is in good agreement with the previous results on the dehydrogenation of cycloalkanes [22,27,37]. It can also be seen that the addition of copper positively affects the selectivity of MCH conversion routes. Indeed, an increase in the copper content leads to a hindering of the side reaction of MCH hydrogenolysis into benzene and methane, thus promoting a significant increase in the selectivity and yield of toluene. It is important to note that this trend appears even at low copper loadings, i.e., for the Cu5Ni95–SiO_2_ and Cu5/Ni95–SiO_2_ catalysts, for which the TOL yield increased by almost 10 mol% compared to the monometallic catalyst. At higher copper loadings, the efficiency of TOL formation became even more pronounced, showing a 1.5–2-fold increase in the TOL yield (ca. 50–60%).

The amount of carbon deposits on the catalysts after catalytic experiments relates well to the TOL selectivity. Indeed, the carbon content for the monometallic Ni–SiO_2_ catalyst and the samples with low copper loadings (Cu5Ni95–SiO_2_ and Cu5/Ni95–SiO_2_) was about 3.9–4.2 wt%, and decreased with an increase in the copper content. For example, for the Cu20Ni80–SiO_2_ and Cu20/Ni80–SiO_2_ catalysts, the carbon contents were 2.4 and 1 wt%, respectively, while for the Cu30/Ni70–SiO_2_ catalyst, the value decreased down to 0.6 wt%. In general, these results show that the pCu-series catalysts exhibited higher tolerance to carbon deposition in the dehydrogenation of MCH than the SG series. The highest TOL yields of 57.8% and 59.6% were observed for the Cu20/Ni80–SiO_2_ and Cu30/Ni70–SiO_2_ catalysts, which corresponded to 70% and 77% selectivity toward toluene and MCH conversion of 82.5% and 77.5%, respectively. Noticeably, noble-metal-based catalysts generally possess high activity and targeted aromatic selectivity (+99%) for MCH dehydrogenation, which is difficult to achieve [9,38]. However, a strong effect of noble-metal-based catalyst deactivation on long-term selectivity has been noticed, e.g., for monometallic Pt catalysts, along with bimetallic Pt–Re and Pt–Pd catalysts [9]. Hence, the development of a low-cost, highly active, and selective dehydrogenation catalyst with a low deactivation rate remains crucial for industrial applications. It is important that among potential non-precious-metal catalysts, the bimetallic Ni–Cu combination appears promising. To the best of our knowledge, such high activity and selectivity levels as reported in the present study have not been achieved for the dehydrogenation of MCH over Ni–Cu-based catalysts so far. In the only work that has reported similar TOL selectivity over Ni–Cu catalysts, [39] the MCH conversion did not exceed 25%. Patil et al. [39] studied catalysts supported on activated carbon cloth (Ni–Cu/ACC), and came to a similar conclusion that a catalyst with a favorable ratio between Ni and Cu (8 wt% Ni + 2 wt% Cu/ACC) has high potential in the dehydrogenation of MCH. Despite the fact that their data were also obtained in a continuous-flow reactor in a flow of hydrogen and argon, the conditions they applied were generally different from those used in the present work. Therefore, a proper comparison of quantitative data from these two studies might prove difficult. Nevertheless, we can confidently assume that the Cu20/Ni80–SiO_2_ and Cu30/Ni70–SiO_2_ catalysts are not inferior to the non-noble nickel-based catalysts reported previously. Based on the fact that the Cu20/Ni80–SiO_2_ catalyst showed a higher MCH conversion (at a close TOL yield) than the Cu30/Ni70–SiO_2_ catalyst, the former system was chosen for further detailed studies.

Table 2 shows that at the same Cu/Ni atomic ratios, the pCu-series catalysts exhibit higher yields and selectivity toward toluene than the corresponding samples in the SG series. This indicates that the method of introduction of copper into the catalyst significantly affects the interaction between its components and, thus, its catalytic action in MCH conversions. Moreover, this clearly demonstrates that the impregnation approach provides a more efficient interaction between copper and nickel than the method used to synthesize the SG series. In order to shed some light on this effect, and to provide a deeper understanding of the relationship between structural features of the catalysts and their catalytic performance, a set of physicochemical methods was applied for the detailed study of the catalysts.

### 3.2. Catalyst Characterization

It is evident that the catalysts from both series possess a high specific surface area (SSA) of ~150–300 m^2^/g_cat_, despite the high Ni loading (about 47–62 wt% for oxidized catalysts, Table 1). This might be explained by the high specific surface area of the Ni-containing precursor used in the heterophase sol–gel synthesis. The scanning electron microscopy images showing the “sponge-like” morphology of this Ni-containing precursor (Figure S1) provide additional evidence for this assumption. Notably, SSA generally decreases with increasing copper content, which is more pronounced in the case of the pCu catalysts. Here, for both series, the samples with a Cu/Ni ratio of 0.05 present an interesting exception. The general trends are not captured in these cases, since the respective SSAs appear lower than those of the catalysts with a 0.1 Cu/Ni ratio. As a possible explanation, the combination of two major factors influencing the SSA behavior can be suggested: On the one hand, in the case where a small amount of copper is introduced to the catalyst, the reducibility of the Ni oxide species becomes thermodynamically favored, resulting in some agglomeration and decreasing the SSA (0.05 Cu/Ni ratio). However, when we add more copper to the catalyst (Cu/Ni ratio exceeding 0.1), oxidized copper readily transforms to the metallic state, and presumably provides a kind of a barrier for the agglomeration of nickel species upon reduction. When performing CO chemisorption measurements, the decreasing CO adsorption was demonstrated with an increase in the Cu content. Table 3 summarizes the data on chemisorption of carbon monoxide at ambient conditions (25 °C, 0.1 MPa) on reduced catalysts.

The amount of CO chemisorbed by the reduced monometallic Ni–SiO_2_ catalyst was 555 μmol/g_cat_, reflecting the number of active surface species. As discussed previously [40], the total amount of chemisorbed CO (in μmol per gram of catalyst) could be considered a reliable parameter for the characterization of the surface area of an active component in high-loaded Ni-based systems. For these catalysts, as well as for the Ni–Cu samples in the present study, the proper calculation of the active component surface area appears to be complicated. This results from the fact that CO chemisorption can proceed at various sites in a different manner. On the one hand, it is considerably affected by the metal loading, the metal particle dispersion, the metal–support interaction, and the presence of impurities [41,42,43]; this leads to different CO adsorption modes, such as linear, bridged, or carbonyl-type adsorption. On other hand, all of these factors can simultaneously take place on the catalyst’s surface, with different adsorption stoichiometry, which further complicates the calculation of the specific surface area of the active component [41]. Moreover, Cu sites can also contribute to the adsorption of CO, although in the case of Cu, the heat of CO adsorption is extremely low compared to Ni [41,44]. For both the pCu and SG series, an increase in the Cu content led to a gradual decrease in the consumption of CO. However, at the same Cu/Ni atomic ratio, the amount of chemisorbed CO was higher for the SG catalyst series. Asedegbega et al. [41] suggested that in the case of the Ni–Cu bimetallic system, a decrease in the heat of adsorption and in the amount of adsorbed CO originated from the introduction of Cu. In the case of catalysts reduced at 350 °C, this was interpreted as a result of the formation of a Ni–Cu solid solution, whereas for catalysts reduced at 500 °C, it was attributed to partial segregation of metallic particles. In contrast, when Cu was introduced into a Ru–Cu system, the variations in the heat of adsorption and the amount of adsorbed CO were negligible, suggesting no formation of solid solutions. Considering these observations, it can be assumed that the more effective formation of Ni–Cu solid solutions occurs in the pCu catalysts, and this could be a reasonable explanation of the lower CO adsorption values observed for catalysts of this series (Table 3).

**Figure 1:**
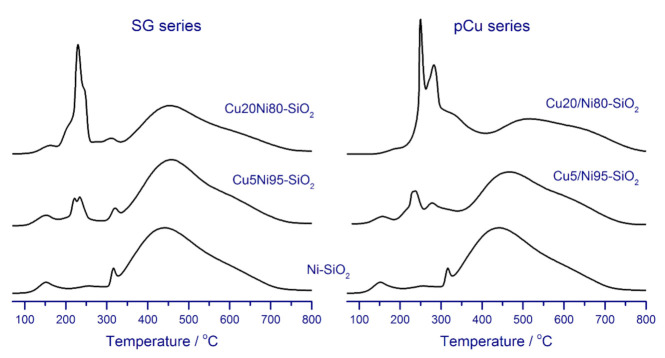
Temperature-programmed reduction profiles of the catalysts.

Investigation of the reducing ability of nickel–copper catalysts and the monometallic catalyst by the temperature-programmed reduction provides additional insights into the interaction of these two metals. As seen in Figure 1, two regions can be distinguished on the TPR curves of the synthesized catalysts: low-temperature, and high-temperature. In the high-temperature region for all catalysts, there is an extended peak at 350–700 °C, which is caused by the reduction of nickel oxide species strongly bound to the support and/or forming nickel silicate structures [29]. In the low-temperature region, the TPR curves exhibit several peaks. A small hydrogen absorption peak at 160–170 °C is usually attributed to the reduction of Ni(III) species formed by oxygen chemisorption on highly dispersed NiO [45]. The peak at 300–320 °C, which is most clearly observed in the case of the monometallic Ni–SiO_2_ catalyst, is explained by the reduction of finely dispersed nickel oxide species weakly bound to the support. These species are reduced at a lower temperature compared to large, well-crystallized NiO particles (400–500 °C).

In contrast to the monometallic sample, the TPR curves of the catalysts modified with copper exhibit several peaks at 220–300 °C. This region corresponds to the reduction of copper oxide species, as well as to the reduction of various forms of nickel oxide, induced by the interaction with copper. It has been shown that the introduction of copper lowers the reduction temperature of Ni^2+^ [45,46,47]. Although the origin of this phenomenon has not yet been fully understood, it is usually associated with the formation of solid solutions of metal oxides. Therefore, a close interaction of NiO and CuO species forming a solid solution is assumed to change the Gibbs energy for the reduction of these forms [48]. Indeed, the molar free energy for the reduction of NiO (−12 kJ mol^−1^ at 25 °C) is higher than that for CuO (−102 kJ mol^−1^ at 25 °C) [49]. Thus, in the case of the formation of a NiO–CuO solid solution, the Gibbs energy for the reduction is expected to have a value between these extremes; therefore, the reduction temperature for nickel species should become lower. As demonstrated by Fedorov et al. [47], the formation of solid solutions of Cu^2+^ in NiO leads to a decrease in the activation energy of nickel oxide reduction by 30–40 kJ mol^−1^; they also found that, although a distortion of nickel oxide lattice leads to the growth of nucleation sites, it cannot lead to a significant change in the activation energy of the reduction process; thus, the authors supposed that a decrease in the activation energy—and, thus, the reduction temperature—of NiO in its solid solutions with Cu^2+^ was due to a change in the chemical state of Ni^2+^ species. In turn, Nagash et al. [50] suggested that alloying nickel with copper increases the covalence of the Ni–O bond, resulting in lower binding energy of Ni and decreasing the reduction temperature observed in their study. In other words, the change in the chemical state of nickel seems to be a reasonable explanation for the decrease in the activation energy and temperature of nickel reduction.

As seen from Figure 1, an increase in the copper content leads to a notable increase in the relative intensities of peaks at 220–300 °C for both catalyst series.

In contrast, the hydrogen consumption in the high-temperature region, which is attributed to the reduction of nickel oxide species that are strongly bound to the support due to the formation of nickel silicate structures, gradually declines. Moreover, this trend is more pronounced for the pCu-series samples, which is evidenced by the large difference between the profiles of Cu20Ni80–SiO_2_ and Cu20/Ni80–SiO_2_. By analogy to the discussion provided for the CO chemisorption data, we believe that these observations indicate a more effective formation of Ni–Cu solid solutions in the pCu catalysts.

**Figure 2:**
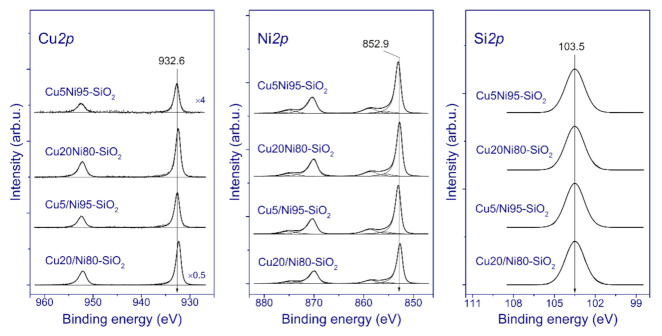
Normalized Cu*2p*, Ni*2p*, and Si*2p* core-level spectra of the reduced catalysts.

The reduced catalysts were studied via XPS; it should be stressed that the catalysts were additionally reduced with H_2_ at 350 °C inside the high-pressure cell connected to the photoelectron spectrometer. Figure 2 presents the Cu*2p*, Ni*2p*, and Si*2p* core-level spectra of the catalysts.

As can be seen, the Si*2p* spectra exhibit a wide symmetric peak corresponding to silicon in the Si^4+^ state. Note that, in this case, it is difficult to determine the chemical state of silicon via XPS. Indeed, according to the literature data, silicon in SiO_2_ and in silicate structures is characterized by similar values of the Si*2p* binding energy at 103.3–103.8 and 103.0–103.5 eV, respectively [51,52,53,54,55]. In contrast, the Ni*2p* spectra undoubtedly indicate that nickel in the reduced catalysts is mainly in the metallic state: the spectra contain two intense narrow peaks at 853.0 and 870.2 eV, corresponding to the Ni*2p_3/2_*–Ni*2p_1/2_* spin–orbital doublet that is typical of metallic nickel [56]. Additional weak peaks at 858 and 875 eV correspond to energy-loss peaks due to plasmon excitations in nickel in the metallic state [56]. One can see that an increase in the copper content leads to a decrease in the Ni*2p*_3/2_ binding energy from 852.9 to 852.7 eV. This effect can be attributed to the formation of a Ni–Cu solid solution in the catalysts. Previously, it was shown that the Ni*2p_3/2_* binding energy of the Ni_x_Cu_1-x_ solid solutions decreases with increasing copper content [57].

The Cu*2p* spectra also consist of two sharp Cu*2p_3/2_* and Cu*2p_1/2_* peaks at approximately 932.6 and 952.4 eV, respectively, due to spin–orbital splitting. The absence of intense shake-up satellites and the Cu*2p_3/2_* binding energy in a range of 932.3–932.6 eV indicate that copper in the catalysts is in the metallic state or in the Cu^1+^ state. There are no Cu^2+^ species, because these are characterized by a higher Cu*2p_3/2_* binding energy. Moreover, the Cu*2p* spectrum of a Cu(II)-based compound contains intense shake up satellites [58]. To distinguish Cu^1+^ from Cu^0^, it is necessary to use the Auger parameter, which is equal to the sum of the Cu*2p_3/2_* binding energy and the position of the maximum of the Cu*LMM* Auger peak on the scale of the kinetic energies of electrons. According to the literature data, the Auger parameters of metallic copper, Cu_2_O, and CuO are 1851.0–1851.4, 1848.7–1849.3, and 1851.4–1851.7 eV, respectively [58,59]. In our case, the Auger parameter is in the range of 1850.9–1851.3 eV for all of the reduced catalysts, which strongly indicates that copper is mainly in the metallic state. Therefore, the reductive treatment of the catalysts in hydrogen results in a complete transformation of nickel and copper species to the metallic state, at least in the near-surface layers of the catalysts.

Relative atomic concentrations (atomic ratios) of elements were estimated for the near-surface layers of the catalysts after additional hydrogen treatment in the high-pressure cell (Table 4).

The relative copper concentration expectedly increases with the copper content for both catalyst series. However, for the pCu catalysts, the surface atomic copper concentrations are higher than those for the SG samples with the same copper loadings. For example, for Cu20/Ni80–SiO_2_, the Cu/Ni atomic ratio is 0.244, which is 2.5-fold higher than that for Cu20Ni80–SiO_2_. This fact correlates with the CO chemisorption data (Table 3), which very likely indicate a higher dispersion of copper species in the pCu series.

**Figure 3:**
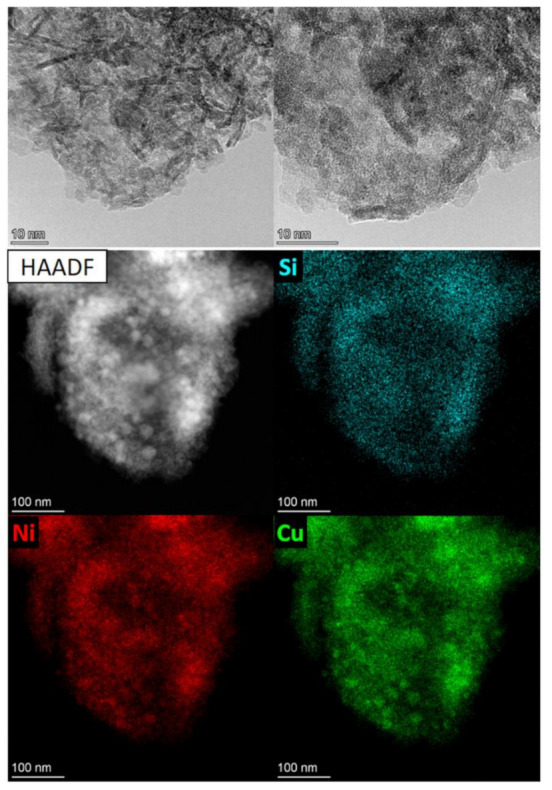
HRTEM images and EDX mapping (in HAADF STEM mode) of the passivated Cu20/Ni80–SiO_2_ catalyst.

The passivated catalysts were studied by HRTEM and EDX (Figures S2–S5). The micrographs of higher resolution and the EDX mapping for the reduced Cu20/Ni80–SiO_2_ catalyst are presented in Figure 3.

The catalysts are characterized by a lamellar (layered) structure consisting of thin oxide–silicate species typical of sol–gel Ni-based catalysts [60]. These species remain after the reduction, which is consistent with the TPR data. It was suggested that the lamellar structure specifically provides the high specific surface area of the sol–gel catalysts and the high nickel dispersion. The EDX analysis implies that all elements (nickel, copper, and silicon) are uniformly distributed throughout the catalysts. In particular, according to the microphotographs, the samples mostly contain highly dispersed species with a size of 2–5 nm. The measured lattice spacings—equal to ~0.24 and 0.21 nm—in combination with the EDX analysis, point to the Ni–Cu oxide nature of these species. Moreover, since the lattice spacing of 0.21 nm is also intrinsic to the metallic nickel, the presence of such species cannot be ruled out either. The possibility of the formation of well-dispersed Ni–Cu solid solutions is also worth considering. The presence of bimetallic Ni–Cu particles of ~10 nm in size, covered with an oxide film, was deduced from the images (Figure S6), in good agreement with the XRD data provided below. Moreover, the possibility of the formation of metal solid solutions was also supported by the fact that, apart from scarce copper particles (Figure 3, HAADF STEM mode), most of the HRTEM images did not reveal copper particles, indicating a high dispersion of copper in these samples (Figures S2–S5). Furthermore, the EDX mapping of the reduced Cu20/Ni80–SiO_2_ catalyst reveals that the signals of Ni and Cu overlap (Figure 3). To further clarify the possibility of the formation of bimetallic Ni–Cu solid solutions, the catalysts were studied via in situ X-ray diffraction and X-ray absorption spectroscopy (see below). In general, the HRTEM study indicated a high dispersion of nickel in both catalyst series, but did not reveal essential differences in their morphology.

**Figure 4:**
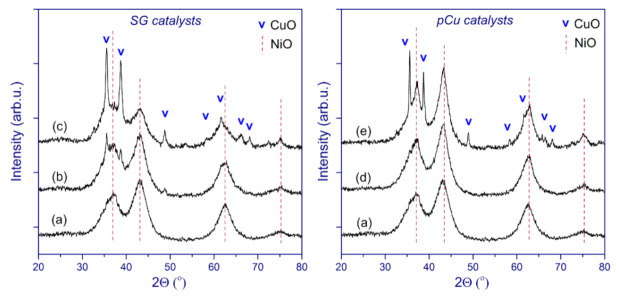
XRD patterns of the catalysts in oxide form: (a) Ni–SiO_2_; (b) Cu5Ni95–SiO_2_; (c) Cu20Ni80–SiO_2_; (d) Cu5/Ni95–SiO_2_; and (e) Cu20/Ni80–SiO_2_.

Figure 4 demonstrates the X-ray diffraction patterns of the catalysts in the oxide state. All of the diffractograms reveal broadened reflections corresponding to NiO (JCPDS no. 471049) and narrow reflections of CuO (JCPDS no. 05-661).

At first sight, despite observable differences in the shape of nickel oxide reflections between the two catalyst series, the size of the coherent scattering domain (CSD) of NiO for the pCu samples was estimated to be ~35 Å, only slightly exceeding the values of ~30 Å for the SG series. Therefore, it can be concluded that the additional calcination procedure after copper nitrate impregnation—which was applied for the pCu catalysts—had little effect on the formation of the nickel oxide phase. It is important to note that the ratio of intensities of the reflections due to CuO and NiO is lower for the pCu catalysts than for the SG catalysts; this is especially noticeable for the low copper content (bulk atomic Cu/Ni = 0.05, Table 1), for which the CuO reflections are much more pronounced for the SG catalysts. At the same time, it was noted that the CSD size of CuO was higher for the pCu catalysts (580 Å for Cu20/Ni80–SiO_2_, in contrast to 260 Å for Cu20Ni80–SiO_2_), which indicates the formation of well-crystallized particles. For the Cu5/Ni95–SiO_2_ sample, the CSD size of CuO could not be estimated, due to relatively low reflection intensity. These data might indicate that the pCu catalysts contain a small fraction of large, well-crystallized copper oxide particles, while the substantial part of copper oxide exists in a highly dispersed state.

**Figure 5:**
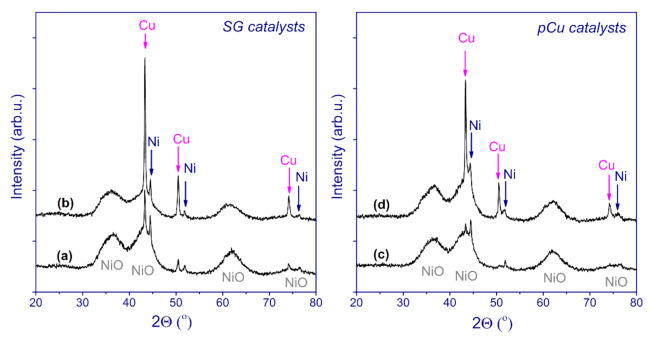
XRD patterns of the passivated catalysts: Cu5Ni95–SiO_2_ (a); Cu20Ni80–SiO_2_ (b); Cu5/Ni95–SiO_2_ (c); and Cu20/Ni80–SiO_2_ (d).

The XRD patterns of the reduced catalysts are presented in Figure 5. First, it could be inferred that the reflections corresponding to the metallic Cu and Ni particles in the powder XRD patterns should partially come from those metal particles well distinguished in the micrographs (Figure 3 and Figure S6). Along with metallic Ni and Cu, all of the catalysts contain residual unreduced NiO, which is consistent with the TPR-H_2_ data. The absence of the reflections corresponding to copper oxide likely suggests that CuO is completely reduced under the conditions used. In general, the conclusions made above for the catalysts in the oxide form are confirmed by the data on the reduced samples. Here, the relative intensity of copper reflections in Cu5Ni95–SiO_2_ is significantly higher than that of those in the XRD pattern of Cu5/Ni95–SiO_2_. A quite similar trend is observed when comparing the Cu20Ni80–SiO_2_ and Cu20/Ni80–SiO_2_ catalysts.

**Figure 6:**
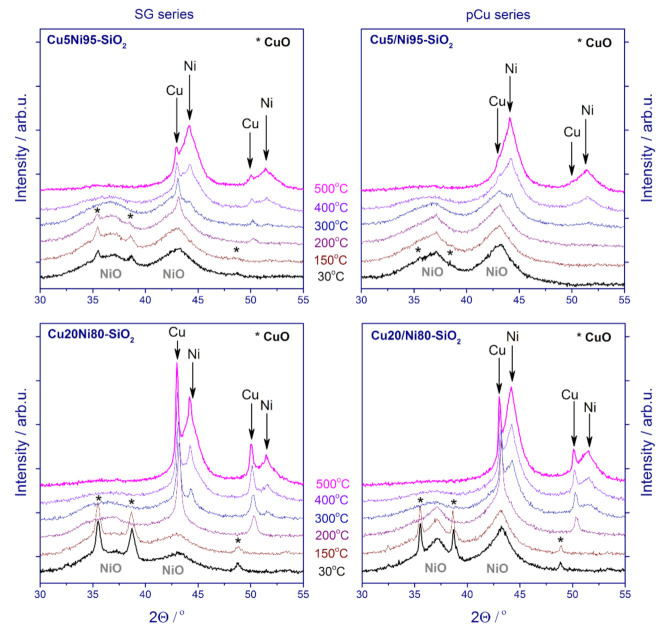
XRD patterns of the catalysts after reduction in situ in the diffractometer chamber.

A detailed study of the phase transformations that occur during the reduction of the catalysts was performed via in situ XRD (Figure 6). The XRD patterns were collected during stepwise heating from room temperature to 500 °C in the 10 vol% H_2_ balanced in an Ar flow. For all of the catalysts, a gradual decrease in the intensity of oxide species reflections was accompanied by the appearance of peaks corresponding to metallic Cu (at 200 °C) and Ni (at 300 °C). Moreover, at 300 °C the relative intensity of Ni reflections significantly increased for the catalysts with the higher copper content (bulk atomic Cu/Ni = 0.23, Table 1). Again, this tendency is more pronounced for the pCu catalysts. These observations are consistent with the TPR-H_2_ data, and provide additional confirmation of the intense reduction of Ni in the presence of Cu as a result of their more efficient interaction. As the reduction temperature increases, the intensity of metallic Ni reflections rises while, conversely, that of NiO reflections decreases, with almost complete disappearance of the NiO phase at 500 °C. The observed differences for the catalysts reduced ex situ (Figure 5) may be caused by the reverse oxidation of finely dispersed metal particles after passivation and contact with air. As a result, a lesser number of metal particles contribute to the corresponding reflections in the diffraction patterns.

Table 5 demonstrates that the lattice parameter of metallic nickel for the as-prepared catalysts (passivated) is in the range of 3.525–3.540 Å; this was deduced from the respective shift of (111) Ni reflections toward lower 2Θ values.

The reference lattice parameter of metallic nickel is 3.523 Å; thus, the observed higher values are assumed to be the result of the formation of Ni–Cu solid solutions. The Cu20/Ni80–SiO_2_ catalyst reveals the highest lattice parameter, which refers to the formation of a solid solution with increased copper content. According to Vegard’s law, the unit cell parameters should change linearly with the composition of a solid solution. This generalization applies to solid solutions formed by random substitution or distribution of ions [61]. Thus, the changes in the unit cell parameters result from the relative sizes of the atoms or ions taking part in a simple substitution mechanism. In the present study, the atomic composition of the Ni_x_Cu_1-x_ solid solutions was roughly estimated based on their relation to the lattice parameters provided by Sinfelt et al. [62]. The respective approximate compositions of Ni_x_Cu_1-x_ are given in Table 5 for the samples of both series with Cu/Ni weight ratios of 5:95 and 20:80. As seen from Table 5, the average atomic compositions of Ni_x_Cu_1-x_ solid solutions estimated using the XRD data for the pCu catalysts correspond well to the atomic ratios embedded in the catalysts. This provides an additional indication of more effective interaction between nickel and copper in the pCu catalysts. It is worth noting that, for the Cu20Ni80–SiO_2_ catalyst, only a small deviation of the lattice parameter of Ni is obvious, reflecting less effective interaction between the two metals compared to its pCu counterpart.

As previously noted, an increase in the copper content leads to a decrease in the contribution of side hydrogenolysis, and to a corresponding significant growth in the selectivity and yield of toluene. This effect is most likely due to the formation of Cu–Ni solid solutions. Indeed, as shown by Sinfelt et al. [62], the specific activity of nickel–copper catalysts as a function of the catalyst composition is quite different for the hydrogenolysis of ethane and the dehydrogenation of cyclohexane. At that, the catalytic activity in the hydrogenolysis of ethane decreased markedly and continuously with an increase in the atomic percentage of Cu in the solid solution, over the whole range of studied compositions. In contrast to the results in the hydrogenolysis of ethane, the catalytic activity in the dehydrogenation of cyclohexane remained insensitive to the composition of a Cu–Ni solid solution over a wide range. Similar conclusions were drawn when testing Ni–Cu/Al_2_O_3_ catalysts in the hydrogenolysis of glycerol to 1,2-propanediol [63]. The authors explained the variation in the catalytic performance from the viewpoint of the ensemble theory; this approach considers that the addition of copper results in a dilution of the active Ni phase, thus decreasing the size of the Ni ensembles available on the active surface. The authors showed that the total amount of products of C–C hydrogenolysis decreased exponentially when the fraction of surface Ni atoms was reduced; this implies that the cleavage of C–C bonds in glycerol requires a special ensemble of ”neighboring” Ni atoms. Another explanation for the influence of the second metal on the catalytic performance is based on the electronic effect [64]. The electronic factor is associated with the modification of the electron density of active species when forming a solid solution or changing the metal particle size. These variations in the electron density led to a change in the adsorption heats of reaction intermediates [39,64]. Nevertheless, it is clear that this factor is inextricably linked to the geometric effect, and that the two cannot be considered separately. By analogy to the above observations, we can confidently assume that the structure-dependent hydrogenolysis observed in the case of MCH in the present study is definitely more sensitive to the formation of Ni–Cu solid solutions than the dehydrogenation of MCH to TOL.

**Figure 7:**
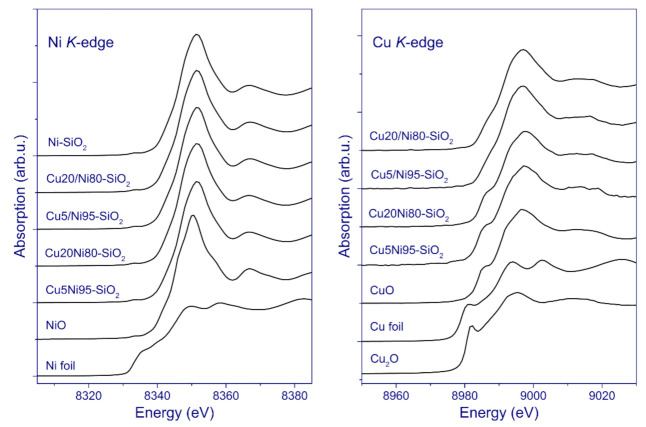
Normalized Ni and Cu K-edge XANES spectra of the catalysts in oxide form, in comparison to the reference spectra of NiO and Ni foil or CuO, Cu_2_O, and Cu foil.

For additional information, X-ray absorption methods were used to study the chemical composition and structure of these catalysts. Ni and Cu K-edge XANES spectra of the catalysts in the oxide state, as well as the spectra of Ni and Cu foil, NiO, Cu_2_O, and CuO, are shown in Figure 7. The K-edge of metallic nickel is at 8333 eV [65]. The XANES spectrum of metallic nickel is characterized by the presence of two peaks of approximately the same intensity at the absorption edge in the region of 8350–8360 eV, as well as the pre-edge feature in the form of a shoulder at 8335 eV (1s→3d transition). In contrast, the spectrum of NiO has an intense peak at the absorption edge in the region of 8350 eV [66,67]. The Ni K-edge XANES spectra of the SG catalysts have a pronounced peak at 8351.0 eV, which is characteristic of NiO. Similar observations are made for the pCu catalysts, for which the Ni K-edge XANES spectra appear almost the same as for the SG catalysts.

The absorption edge for metallic copper is at 8979 eV [65]. The Cu K-edge XANES spectra of the samples from the SG series have a similar shape close to the reference CuO spectrum, for which an intense peak at 8997 eV and a pronounced shoulder at 8985 eV are observed. This suggests that copper in these catalysts is in the Cu^2+^ state, mainly in the form of CuO. At the same time, the Cu K-edge XANES spectra of the pCu catalysts differ from those of the SG catalysts. Apparently, the spectra of pCu series have an intense peak at the absorption edge in the region of 8997.0 eV; however, the shoulder at approximately 8985 eV is not well pronounced; this suggests that, along with the CuO state, the pCu catalysts contain copper in a state that is different from pure CuO, Cu_2_O, or metallic copper. Presumably, a more effective interaction between species of copper oxide and nickel oxide might be responsible for the shape of the Cu K-edge XANES spectra, which is consistent with the TPR-H_2_ and XRD data.

**Figure 8:**
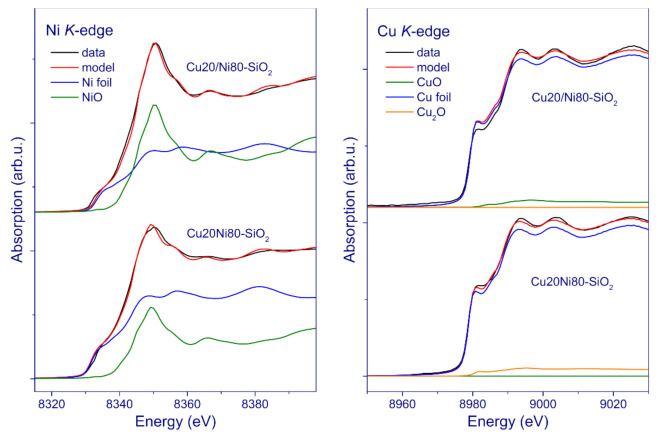
Ni and Cu K-edge XANES spectra of the Cu20Ni80–SiO_2_ and Cu20/Ni80–SiO_2_ catalysts obtained in situ during reduction in H_2_ flow at 400 °C, and fitting the catalyst spectra by a linear combination of NiO and Ni foil or CuO, Cu_2_O, and Cu foil spectra.

The reduction of the Cu20Ni80–SiO_2_ and Cu20/Ni80–SiO_2_ catalysts was examined in situ in a hydrogen flow at 400 °C. Figure 8 shows the linear combination fitting (LCF) for the Ni and Cu K-edge XANES spectra of the samples, using the reference spectra of Ni and NiO or Cu, Cu_2_O, and CuO phases, respectively. The LCF results for the Cu20Ni80–SiO_2_ catalyst reveal that approximately 70% of nickel is in the metallic state, while the rest is in the NiO state. For the Cu20/Ni80–SiO_2_ sample, LCF indicates a roughly equal contribution of metallic and oxidized states of nickel. LCF of the Cu K-edge XANES spectra showed that copper in the studied catalysts is preferably in the metallic state, and only 1–5% of total copper atoms are in an oxidized state. At that, the data obtained indicate the incomplete reduction of nickel in both catalysts, which is consistent with the TPR results, pointing to the continuous reduction of nickel up to 700 °C. At the same time, the XRD investigation showed only a very small amount of nickel oxide particles under reduction in situ. These results provide evidence that there are highly dispersed species of nickel oxide that exist presumably in the form of silicate-like structures, which cannot be detected by XRD, but are revealed using X-ray absorption spectroscopy. Therefore, XAS appears quite relevant in this case for providing additional insights into the structural features of the studied catalysts.

By analogy to most of the methods applied in this work, the X-Ray absorption study provided additional evidence that, in the case of impregnation, copper is more evenly distributed in the catalyst (the pCu series). This means a more efficient interaction between the two metals, in contrast to the approach based on mixing solid precursors (the SG series), and exerts a tangible influence over the catalyst activity and selectivity in the dehydrogenation of MCH.

## 4. Conclusions

A new type of Ni-based catalyst, synthesized via the heterophase sol–gel technique and additionally modified with a second metal (Cu), has been studied. A special focus has been placed on the effects of the copper-to-nickel ratio and the method of introduction of copper on the activity and selectivity of the catalysts in the dehydrogenation of MCH. Two approaches to copper introduction have been realized: simultaneous introduction of Ni and Cu from their solid precursors (SG), and wetness impregnation of a sol–gel Ni–SiO_2_ sample with an aqueous solution of copper nitrate (pCu). The monometallic Ni-based catalyst predictably possessed the highest affinity for side reactions (hydrogenolysis) when compared with the bimetallic catalysts. It was observed that side reactions subside as the copper-to-nickel ratio in the catalyst increases to 20:80. The pCu catalysts, which were obtained via wetness impregnation of a high-loaded Ni–SiO_2_ system with a Cu solution, appear to be the most promising.

When studying the catalysts in the oxide state, it was shown that a substantial proportion of the copper oxide in the pCu series exists in a highly dispersed state, and that the interaction of species of copper oxide and nickel oxide is generally more efficient in this case. In a hydrogen-containing atmosphere, the intense reduction of Ni in the presence of Cu was detected by means of XRD in situ, consistent with the TPR-H_2_ data. According to in situ XAS study, in the reduced catalysts, the highly dispersed species of nickel oxide are preserved in the form of silicate-like structures, although they may not be visible in XRD. At the same time, the XPS measurements indicate that during the reduction, nickel and copper species in the near-surface layer are completely transformed into the metallic state. Importantly, the surface atomic concentrations of copper in the pCu catalysts are higher than those in the SG samples with the same copper loadings. These data indicate that copper species in the reduced pCu catalysts are more highly dispersed than those in the SG catalysts, which correlates well with the data on CO chemisorption, XRD, and XAS. Moreover, the XRD study clearly demonstrated that in the case of the pCu series, the atomic composition of the Ni_x_Cu_1-x_ solid solutions was close to the Cu/Ni ratio inherent in the catalyst. These data allow us to conclude that the impregnation approach leads to a more effective interaction between the two metals, resulting in the formation of a Ni_x_Cu_1-x_ solid solution with higher copper content. We believe that these features ensured the higher activity of the pCu series in the dehydrogenation of MCH and the selectivity toward TOL, which makes them an interesting alternative to the catalysts studied so far.

## Figures and Tables

**Table 1:** Elemental composition of the catalysts in oxide form, and specific surface areas (SSA) of the passivated catalysts.

Catalyst ^a^	Composition ^d^, wt%			Atomic Cu/Ni Ratio	Atomic Si/Ni Ratio	SSA ^e^, m^2^/g_cat_
	Cu	Ni	Si			
SG series ^b^						
Ni–SiO_2_	-	62.3	9.7	-	0.33	294
Cu5Ni95–SiO_2_	3.0	59.3	9.7	0.05	0.34	163
Cu10Ni90–SiO_2_	6.3	56.1	9.7	0.1	0.36	293
Cu20Ni80–SiO_2_	12.5	49.9	9.7	0.23	0.41	259
pCu series ^c^						
Cu5/Ni95–SiO_2_	3.3	59.9	9.3	0.05	0.33	234
Cu10/Ni90–SiO_2_	6.4	57.4	8.9	0.1	0.33	262
Cu20/Ni80–SiO_2_	13.0	52.2	8.1	0.23	0.33	235
Cu30/Ni70–SiO_2_	19.9	46.7	7.3	0.4	0.33	157

^a^ Numbers in the catalyst notation correspond to the percentage of metal relative to the total metal loading (wt%). ^b^ Catalyst series obtained via mixing of water-insoluble metal precursors (Cu and Ni) with ES. ^c^ Catalyst series obtained via wetness impregnation of Ni–SiO_2_ with the Cu-containing precursor. ^d^ The composition was determined by X-ray fluorescence analysis. ^e^ Specific surface area was determined via the Brunauer–Emmett–Teller method, using nitrogen adsorption isotherms.

**Table 2:** Catalyst performance in the dehydrogenation of MCH at 325 °C.

Catalyst	MCH Conversion, mol.%	Selectivity to TOL, mol.%	Yield of TOL, mol.%
SG series			
Ni–SiO_2_	81	40	31
Cu5Ni95–SiO_2_	76	49	37
Cu10Ni90–SiO_2_	81	52	42
Cu20Ni80–SiO_2_	87	56	49
pCu series			
Cu5/Ni95–SiO_2_	75	53	40
Cu10/Ni90–SiO_2_	88	57	50
Cu20/Ni80–SiO_2_	83	70	58
Cu30/Ni70–SiO_2_	78	77	60

**Table 3:** Chemisorption of CO for the catalysts reduced in situ at 400 °C.

Catalyst	CO Uptake, μmol/g_cat_
SG series	
Ni–SiO_2_	555
Cu5Ni95–SiO_2_	449
Cu10Ni90–SiO_2_	421
Cu20Ni80–SiO_2_	385
pCu series	
Cu5/Ni95–SiO_2_	384
Cu10/Ni90–SiO_2_	358
Cu20/Ni80–SiO_2_	266
Cu30/Ni70–SiO_2_	213

**Table 4:** Relative atomic concentrations of Ni and Cu in the near-surface layers of the reduced catalysts, as determined by XPS.

Catalyst	Ni/Si	Cu/Si	Cu/Ni
Cu20/Ni80–SiO_2_	0.45	0.11	0.244
Cu5/Ni95–SiO_2_	0.56	0.04	0.071
Cu20Ni80–SiO_2_	0.62	0.06	0.097
Cu5Ni95–SiO_2_	0.61	0.01	0.016

**Table 5:** Lattice parameter of metallic Ni and Ni_x_Cu_1-x_ composition estimated using the data provided in [62] for passivated catalysts.

Catalyst	Lattice Parameter of Ni, Å	Cu/(Cu + Ni) Atomic (XRF)	Ni_x_Cu_1-x_ Composition (XRD)
Cu5Ni95–SiO_2_	3.526	0.046	Ni_0.95_Cu_0.05_
Cu20Ni80–SiO_2_	3.525	0.188	Ni_0.96_Cu_0.04_
Cu5/Ni95–SiO_2_	3.528	0.048	Ni_0.93_Cu_0.07_
Cu20/Ni80–SiO_2_	3.540	0.187	Ni_0.81_ Cu_0.19_

## Data Availability

Not applicable.

## Supplementary Materials

The following are available online at https://www.mdpi.com/article/10.3390/nano11082017/s1: Figure S1: SEM image of nickel(II) carbonate basic hydrate (the nickel precursor used in the sol–gel synthesis) without any treatment (A) and after coating with gold film (B); Figure S2: HRTEM images and representative EDX spectrum of ex situ reduced Cu20Ni80–SiO_2_ catalyst; Figure S3: HRTEM images and representative EDX spectrum of ex situ reduced Cu20/Ni80-SiO_2_ catalyst; Figure S4: HRTEM images and representative EDX spectrum of ex situ reduced Cu5Ni95–SiO_2_ catalyst; Figure S5: HRTEM images and representative EDX spectrum of ex situ reduced Cu5/Ni95–SiO_2_ catalyst; Figure S6: HRTEM image of ex situ reduced Cu20/Ni80–SiO_2_ catalyst: metallic Ni-enriched (Ni–Cu) particle with an oxide cover.
