# Supplementary material for: Ni–Cu High-Loaded Sol–Gel Catalysts for Dehydrogenation of Liquid Organic Hydrides: Insights into Structural Features and Relationship with Catalytic Activity
Source: Nanomaterials (Basel). 2021 Aug 6;11(8):2017. doi: 10.3390/nano11082017 (PMC8398823; doi:10.3390/nano11082017)
Supplement: Supplementary file 1 [file nanomaterials-11-02017-s001.zip › nanomaterials-1322400-supplementary.pdf]

nanomaterials

Article

Ni–Cu High-Loaded Sol–Gel Catalysts for Dehydrogenation of Liquid Organic Hydrides: Insights into Structural Features and Relationship with Catalytic Activity

Yuliya Gulyaeva *, Maria Alekseeva (Bykova) *, Olga Bulavchenko, Anna Kremneva, Andrey Saraev, Evgeny Gerasimov, Svetlana Selishcheva, Vasily Kaichev and Vadim Yakovlev

Federal Research Center Boreskov Institute of Catalysis, Akad. Lavrentiev Ave. 5, 630090 Novosibirsk, Russia; obulavchenko@catalysis.ru (O.B.); kremneva@catalysis.ru (A.K.); asaraev@catalysis.ru (A.S.); gerasimov@catalysis.ru (E.G.); svetlana@catalysis.ru (S.S.); vvk@catalysis.ru (V.K.); yakovlev@catalysis.ru (V.Y.)

* Correspondence: gulyaeva@catalysis.ru (Y.G.); bykova@catalysis.ru (M.A.)

Field Emission Scanning Electron Microscopy

Morphology of the nickel precursor used in the sol-gel synthesis was analyzed using a Field emission scanning electron microscope FESEM, HITACHI SU-8230.

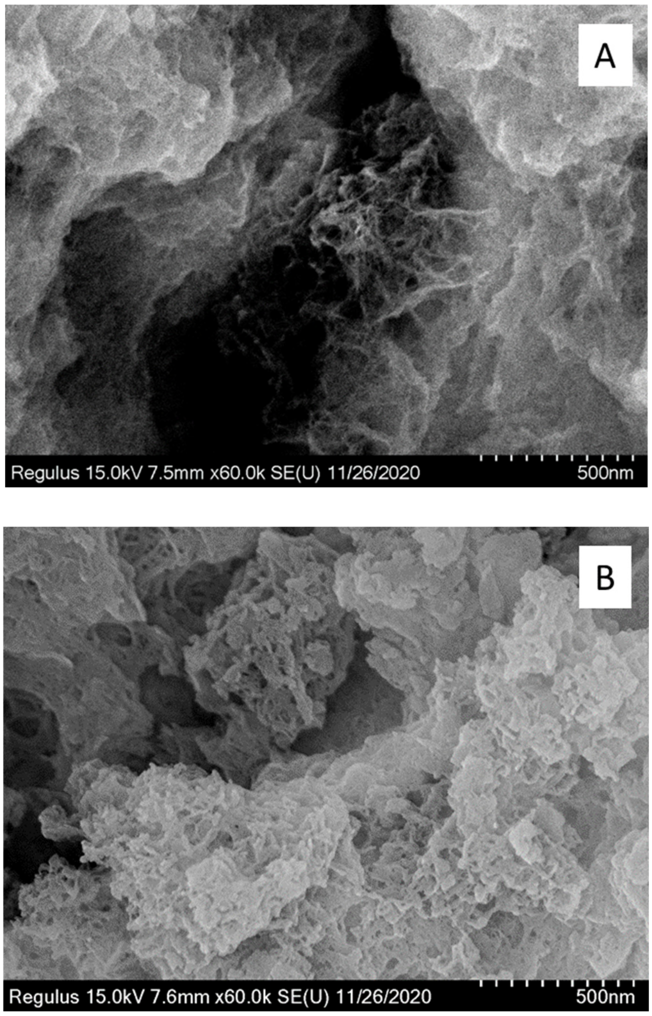

Figure S1. SEM image of nickel(II) carbonate basic hydrate (the nickel precursor used in the sol-gel synthesis) without any treatment (A) and after coating with gold film (B).

High Resolution Transmission Electron Microscopy (HRTEM)

HRTEM images were obtained using a JEM-2010 (JEOL Ltd., Japan, lattice-fringe resolution 0.14 nm) electron microscope operated at an accelerating voltage of 200 kV. The microscope was equipped with an energy-dispersive X-ray (EDX) XFlash spectrometer (Bruker, Germany) with Si-detector and an energy resolution of 130 eV. The samples for the HRTEM study were prepared on a holey carbon film mounted on an aluminum grid by the ultrasonic dispersing of the catalyst suspension in ethanol.

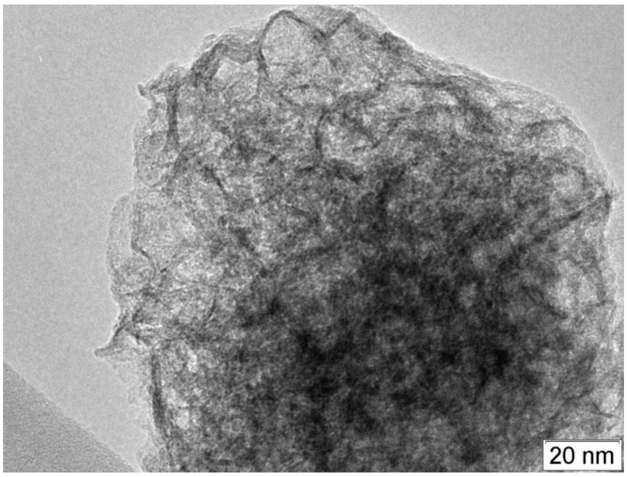

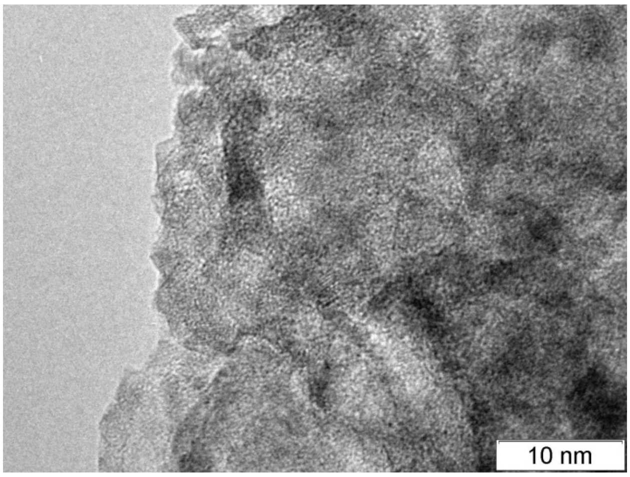

(**a**) (**b**)

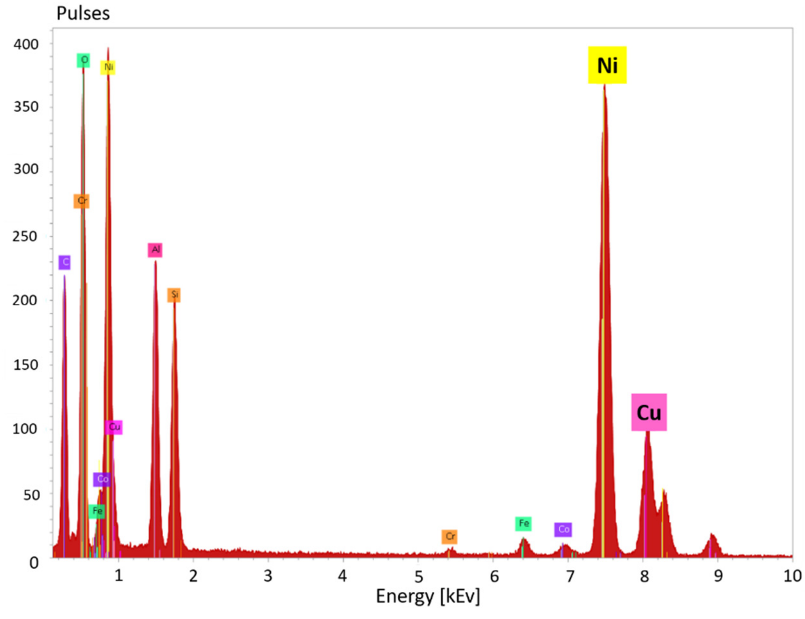

(**c**)

Figure S2. HRTEM images (**a**,**b**) and representative EDX spectrum (**c**) of ex situ reduced Cu20Ni80-SiO_2 catalyst.

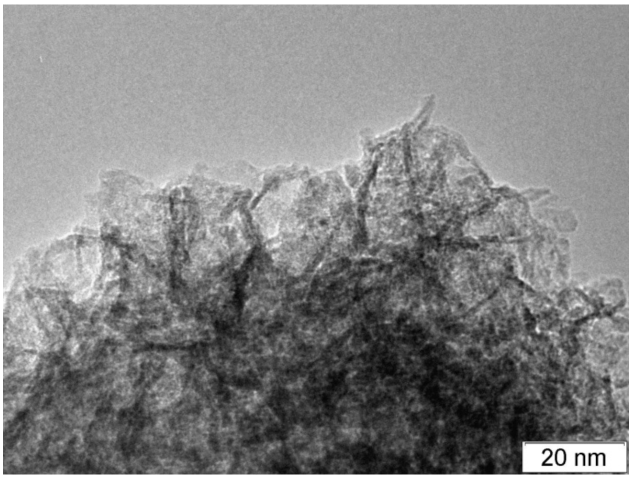

(**a**)

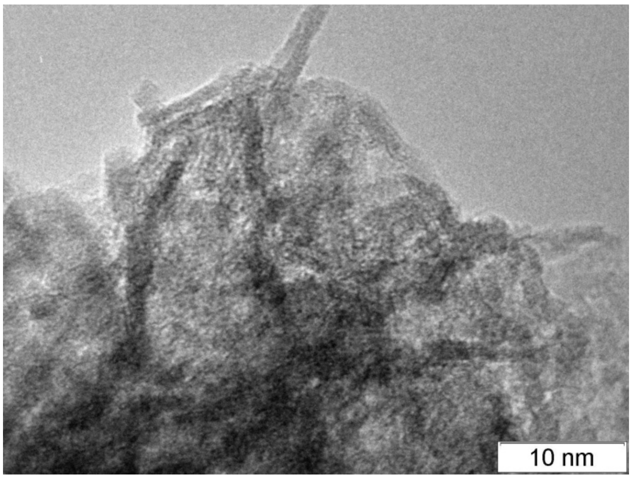

(**b**)

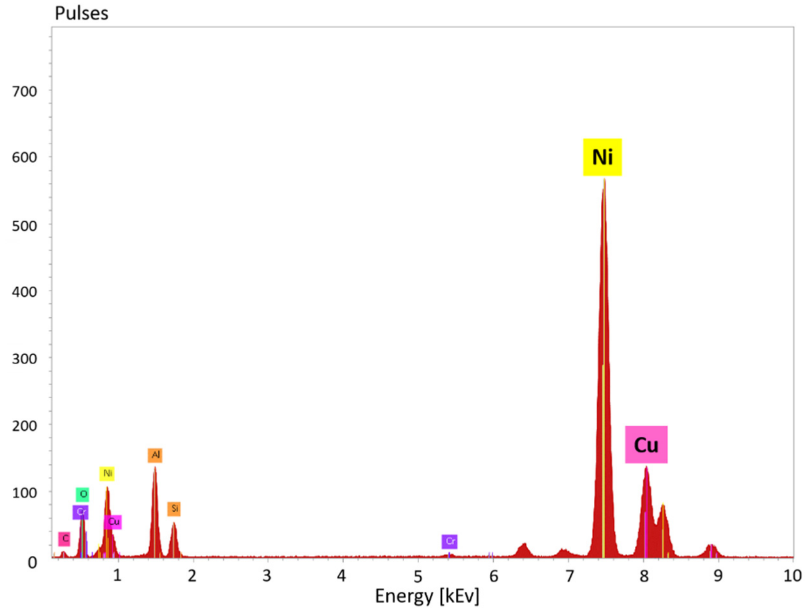

(**c**)

Figure S3. HRTEM images (**a**,**b**) and representative EDX spectrum (**c**) of ex situ reduced Cu20/Ni80-SiO_2 catalyst.

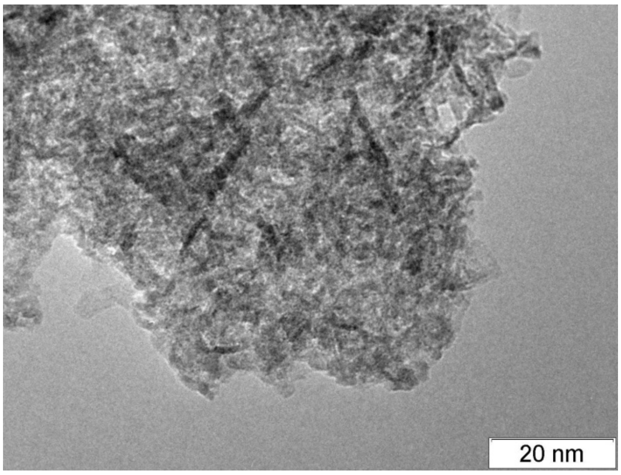

(**a**)

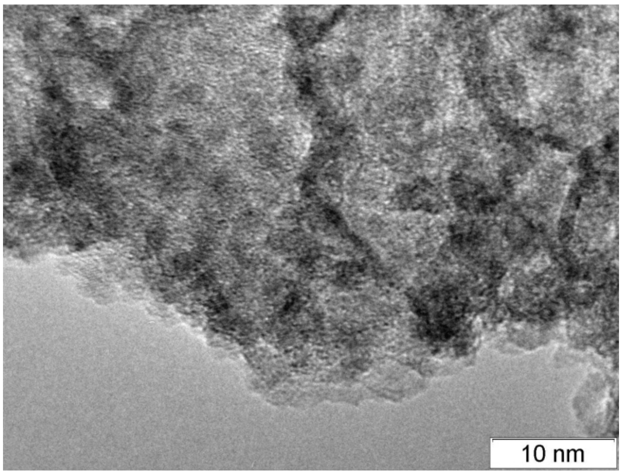

(**b**)

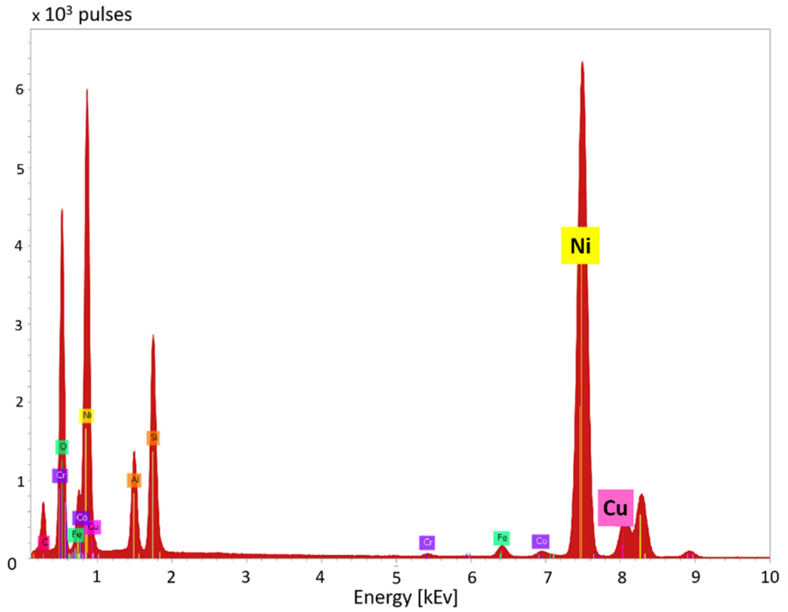

(**c**)

Figure S4. HRTEM images (**a**,**b**) and representative EDX spectrum (**c**) of ex situ reduced Cu5Ni95-SiO_2 catalyst.

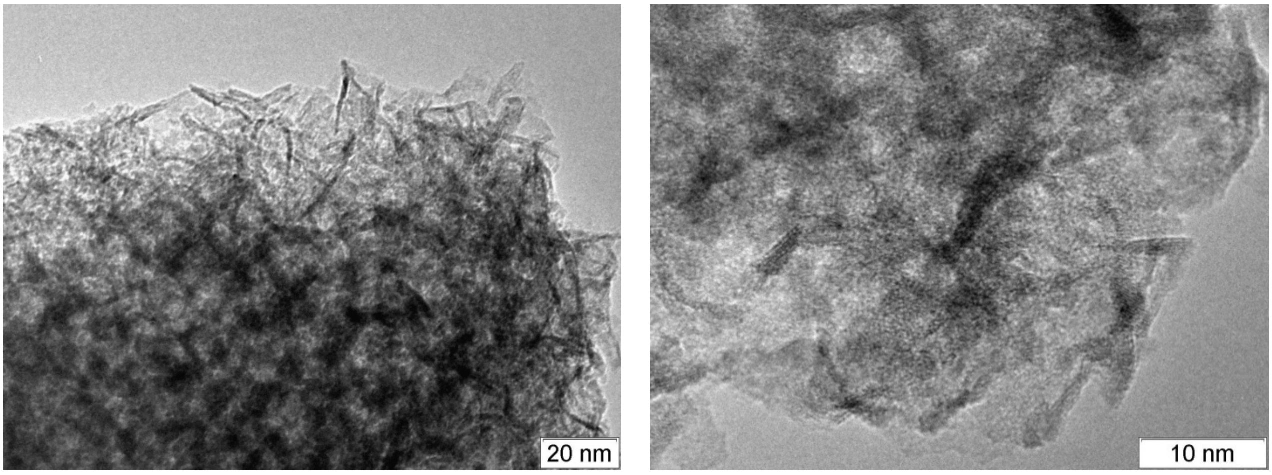

(**a**) (**b**)

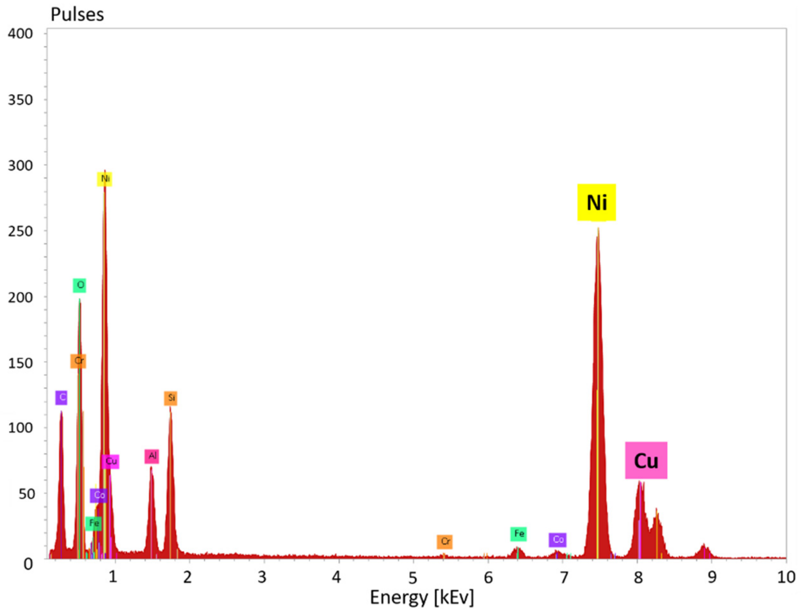

(**c**)

Figure S5. HRTEM images (**a**,**b**) and representative EDX spectrum (**c**) of ex situ reduced Cu5/Ni95-SiO_2 catalyst.

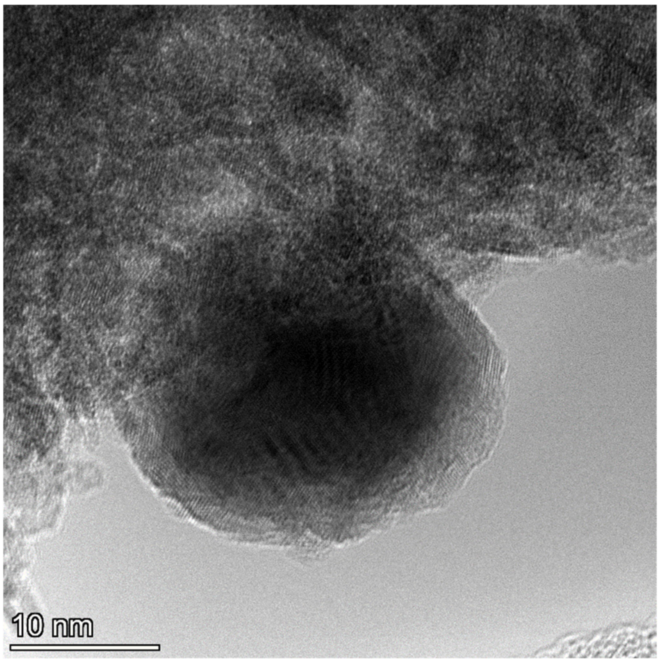

Figure S6. HRTEM image of ex situ reduced Cu20/Ni80-SiO_2 catalyst: metallic Ni-enriched (NiCu) particle with an oxide cover.
